# Reproducibility and Potential for Input Reduction for Torque Teno Virus DNA Quantification in Rheumatoid Arthritis

**DOI:** 10.3390/medsci14040467

**Published:** 2026-08-08

**Authors:** Paul Studenic, Chaimae Akile, Claudia Anna Hana, Daniela Sieghart, Thi Lan Vi Tran, Josef Baliko, Francois Bonnay, Souzi Makri, Daniel Aletaha, Mariet C. W. Feltkamp

**Affiliations:** 1 Division of Rheumatology, Department of Internal Medicine III, Medical University of Vienna, 1090 Vienna, Austriajosef.baliko@meduniwien.ac.at (J.B.); francois.bonnay@meduniwien.ac.at (F.B.);; 2 LUCID Department of Medical Microbiology & Infection Prevention, Leiden University Medical Center, 2300 RC Leiden, The Netherlandsm.c.w.feltkamp@lumc.nl (M.C.W.F.); 3 Cyprus League of People with Rheumatism, Nicosia 1056, Cyprus

**Keywords:** torque teno virus, rheumatoid arthritis, rheumatology, reliability, reproducibility, smallest detectable difference, intra-class correlation

## Abstract

**Background/Objectives**: Torque teno virus (TTV) is small non-pathogenic virus, currently under investigation as a potential biomarker to monitor immunocompetence in patients with rheumatoid arthritis (RA) receiving various immunosuppressive therapies. To allow for large-scale assessment of existing pan-European RA cohorts, minimal specimen (serum) input for TTV DNA detection through quantitative PCR (qPCR) needs to be identified in relation to the type and quantity of immunosuppression. **Methods**: TTV qPCR analysis was performed by measuring 1:1, 1:2, 1:4 and 1:8 dilutions of 278 human serum samples derived from patients with RA under different therapies. The smallest detectable difference (SSD) as well as the intra-class correlation (ICC) were assessed. The reproducibility of results was determined by measuring an additional 19 sample duplicates. **Results**: RA patient sera showed a mean TTV viral load equivalent of 2.9 log10 genome copies/mL (SD ± 1.47). A total of 32 samples (11.6%) were negative for TTV; 54 (19.6%) were below 2.4 log10, the manufacturer’s lower limit of quantification; 44 (16%) were between 2.4 and 3.0 log10; and 145 (52.7%) showed a high viral load above 3.0 log10. Dilution and replication experiments showed a high stability and reproducibility of TTV measurements (overall ICC: 0.95 (95%CI: 0.951 to 0.966)). TTV differed between undiluted and 4-fold- as well as 8-fold-diluted samples (1 vs. 2: *p* = 0.993; 1 vs. 4: *p* < 0.001; 1 vs. 8: *p* < 0.001), aligned with decreasing ICC and less sensitivity. RA disease activity did not influence reproducibility. **Conclusions**: Quantitative TTV DNA detection in serum samples from RA patients yields highly reproducible results, including 2-fold-diluted serum samples.

## 1. Introduction

Torque teno virus (TTV), first identified in 1997, is a circular single-stranded DNA virus belonging to the anellovirus family and is characterized by considerable genetic diversity. TTV is prevalent in the majority of humans, largely independent of the presence of any associated pathology and, according to initial studies, equally prevalent among individuals with rheumatic disorders and those deemed healthy [1,2]. The virus demonstrates a propensity for replication within T lymphocytes and granulocytes; however, the specific cellular receptor facilitating TTV entry remains unidentified [3,4]. TTV is classified as an orphan virus capable of inducing chronic infections that often lack overt clinical manifestations. Nevertheless, its widespread occurrence and persistent nature position it as a potential biomarker for assessing immune status. TTV quantification can be efficiently performed through real-time Polymerase Chain Reaction (PCR) techniques in plasma or other biological specimens and is not influenced by existing antiviral therapies.

In human research, TTV has been utilized to evaluate immune functionality [5]. Various investigations underscore the load of TTV in patients as a prognostic indicator for their immunological capacity. TTV serves as a surrogate marker for the immune system within the context of individuals following stem cell transplantation [5,6]. An elevation in TTV loads and prevalence has been documented across multiple transplant specialties, ranging from hepatic to pulmonary to renal transplantation [7,8,9]. Notably, the variations in TTV load were found to be contingent upon the specific immunosuppressive agents employed against T-cell targets [10]. TTV load was correlated with inadequate immunosuppression, an increased likelihood of allograft dysfunction, heightened immunosuppressive therapy, and consequently, an elevated risk of infections in individuals post-solid organ transplantation [11]. Particularly within the realm of renal transplant recipients, certain TTV load thresholds corresponded with favourable clinical outcomes [12,13,14]. In summary, patients exhibiting elevated TTV loads were found to be at a greater risk for infections, whereas those with diminished TTV load were more susceptible to transplant rejection.

The potential role of TTV in rheumatology is poorly explored, although immunomodulation constitutes the main treatment approach. In rheumatoid arthritis (RA), a chronic inflammatory joint disorder characterized by principal clinical manifestations such as swelling and tenderness, biomarker-driven treatment selection and disease monitoring are still inadequately addressed. An essential objective in the therapeutic management of RA is to facilitate the attainment of remission for affected individuals [15,16,17]. Remission is closely correlated with radiographic stability, optimal functional capacity, and enhanced quality of life [18]. In contemporary clinical environments, disease-modifying anti-rheumatic drugs (DMARDs) are employed and prescribed in accordance with the principles of treat-to-target and in alignment with the European Alliance of Associations for Rheumatology (EULAR) management recommendations for RA [15,19]. These methodologies have represented a pivotal advancement in the treatment paradigm for patients with RA. Nonetheless, these strategies cannot be construed as personalized medicine, as there is a paucity of reliable methods at the commencement of treatment to predict the efficacy of a specific DMARD. Presently, no biomarkers are available that enable the evaluation of whether DMARDs exert a balanced and adequate immunomodulatory effect, aside from the observed alterations in disease activity as quantified by composite scoring systems.

TTV has been investigated as a marker of immunosuppression over the past decade in several scenarios including transplant rejection prevention [20]. Ultimately, TTV has been previously employed as a prognostic biomarker in two investigations pertaining to RA patients. In a randomized-controlled trial that assessed methotrexate-insufficient responders receiving either tumour necrosis factor inhibitors (TNFi), Rituximab, abatacept, or tocilizumab, the study evaluated predictors of therapeutic response to these four distinct mechanisms of action, revealing that TTV load exhibited an elevation during treatment with TNFi, abatacept, and rituximab [21]. The outcomes associated with TNFi were consistent with those observed in an observational study [22]. Furthermore, within the confines of this trial, TTV load measured three months post-initiation of treatment served as an indicator regarding the probability of response. However, knowledge about TTV replication under different mechanisms of action is limited. The influence of pretreatments on differences in TTV replication during DMARD treatments is unknown, similar to other covariates like age, disease duration, disease activity level, potential comorbidities or comedications. In healthy populations, TTV is increasing with age, and a higher TTV load has been found in males [23]. Whether certain compounds particularly influence TTV, or TTV load would represent a generic estimate of immunosuppression, is unclear and necessitates interdisciplinary collaboration of different stakeholders.

Currently, the analysis of the TTV in human serum or plasma samples is performed on undiluted serum, for instance, 200 µL (microliters) per measurement. No data exist on using less specimen volume while maintaining test performance. In the context of efficient biobank and sample management, it is imperative to utilize the minimal volume necessary for each analysis to garner favourable responses and validation from biobank executives, thereby facilitating the successful acquisition of reliable biomaterial and clearly defined research objectives. Beyond biobanked samples, material availability might be limited in different clinical scenarios, like pediatric cases or umbilical cord blood, which represent utmost value.

Our objectives were to assess the consistency of TTV measurements (reproducibility) and to conduct a series of dilution tests on samples from the institutional biobank in order to ascertain the minimum volume and maximum dilution requirements when soliciting collaboration to contribute towards sample collection.

## 2. Materials and Methods

### 2.1. Patient Selection and Sample Storage

Patients with a confirmed diagnosis of RA in long-term follow-up care at our outpatient clinic for rheumatology and included in our prospective observational registry were preselected. Within our registry, clinical and treatment characteristics of the patients’ journeys are documented longitudinally [24,25]. Out of these, 128 patients with RA (also fulfilling the 2010 ACR/EULAR classification criteria for RA [26]) treated with DMARDs of different modes of actions or without DMARD treatment for at least 6 months were matched with samples in the institutional biobank. The selection should encompass a representation of all currently available biological DMARDs (bDMARDs), directed against IL6-R (e.g., tocilizumab), CTLA-4 (abatacept), TNF (e.g., infliximab) and CD20 (rituximab), as well as conventional synthetic DMARDs (csDMARDs). Two serum samples were extracted from a patient’s respective treatment segment—one sample during a state of moderate to high disease activity (clinical disease activity index, CDAI > 12), and one sample during low disease activity or remission (CDAI < 8)—to ensure heterogeneity of different states of RA disease activity and homogeneity in treatment exposure. In addition, a set of 19 duplicate samples derived from randomly selected RA patients were used to study reproducibility of TTV measurements. Patient sample characteristics as well as duplication of serum samples were not revealed to the test laboratory. This purposive selection was designed to represent all relevant DMARD modes of action and to contrast high and low/remission disease-activity states within the same treatment exposure, thereby spanning the analytical measurement range required for a reliability study.

Biomaterial was processed and stored until analysis according to standard operating procedures by the Medical University Vienna biobank, a central facility included in a certified quality management system [27]. Serum samples were stored in 400 µL aliquots at −80 degrees for up to 18.1 years (median: 12.5; IQR: 5.2; min: 1) and did not have more than one freeze–thaw cycle. TTV is a small, non-enveloped, circular single-stranded DNA anellovirus that is physicochemically robust, and anellovirus DNA is considered stable in serum under standardized ultra-low-temperature storage with no more than a single freeze–thaw cycle [28,29]. To assess storage duration as a potential factor for TTV load, a Spearman correlation between storage time and TTV load was performed.

The study was conducted in accordance with the Declaration of Helsinki and approved by the Ethics Committee of the Medical University of Vienna (for clinical data: protocol code 1448/2019, date of approval 15 June 2019; for biobank: protocol code 1075/2021, date of approval 14 June 2021; for data analyses 2002/2014: date of initial approval 1 April 2015). Informed consent was obtained from all subjects involved in the study.

### 2.2. Torque Teno Virus DNA Extraction and Quantification

A total of 278 serum samples were included in the analyses. The sample concentration prior to DNA extraction was reduced stepwise, including a standard measurement using 200 µL of serum (undiluted), and measurements using 100 µL, 50 µL, and 25 µL of serum, corresponding to a dilution factor of 2, 4, and 8, respectively. Diluted samples were supplemented with 1.0 TE buffer to obtain a total sample volume of 200 µL. Finally, 10 µL of internal PCR control 2 (IC2; see below) was added to all samples, resulting in a final extraction volume of 210 µL. DNA was extracted using the MagNa Pure 96 System (Roche Diagnostics, Rotkreuz, Switzerland) and eluted in 100 µL of buffer. For TTV quantification, 15 µL of R33 was mixed with 10 µL of extracted DNA. Viral DNA amplification through the TTV R-GENE^®^ PCR kit was performed using the CFX96 Real-Time PCR Detection System (Bio-Rad, Hercules, CA, USA) with the following cycling conditions: 15 min at 95 °C, followed by 45 cycles of 10 s at 95 °C and 40 s at 60 °C. The undiluted 200 µL input corresponds to the serum volume recommended by the assay manufacturer and to routine testing practice, whereas the 100, 50 and 25 µL conditions represent stepwise 2-, 4- and 8-fold reductions selected for pragmatic biobank and sample-conservation reasons [30,31].

The TTV R-GENE^®^ kit utilizes real-time PCR technology for the detection and quantification of the TTV DNA genome. This amplification and detection step employs the 5′ nuclease hydrolysis probe technique, utilizing a ready-to-use mix containing dNTPs, MgCl_2_, amplification buffer, Taq Polymerase, and specific TTV primers and probes, as well as primers and probes for the internal control, according to the manufacturer’s protocol [30]. The primers are designed to amplify the 5′-UTR region gene, yielding a 128-base-pair fragment. Finally, the system validly quantifies the TTV viral load within a range of 250 copies/mL (c/mL) to 1.0 × 10^9^ c/mL (2.4 log10 to 9.0 log10 c/mL), with results expressed in c/mL of the sample. Following this, log 2.4 c/mL is regarded as the official cut-off for the lowest level of quantification (LLoQ), meaning that viral load might still be detected below, but results might be less reliable.

### 2.3. Data Analyses

For assessing the test–retest reproducibility of TTV quantification, we conducted intra-class-correlation (ICC) analysis in a two-way mixed model with absolute agreement [32]. The coefficient ranges from 0 to 1, where values above 0.9 are considered excellent, 0.75 to 0.9 as good, 0.5 to 0.75 as moderate, and below 0.5 as poor [33,34]. Sample size for the reliability analyses was considered in terms of the precision of the ICC estimate: for an anticipated ICC of approximately 0.9 with two measurements per specimen, a size on the order of 15–20 subjects yields a 95% confidence interval of a width compatible with demonstrating excellent reliability [35,36]. To reach a more practical cut-off for measurement reliability, we calculated the smallest detectable difference (SDD), based on the method of Bland–Altman and its respective plot for visualization [37]. The SDD reflects how large a difference must be to be confident that it is beyond measurement noise, but the difference observed between the two tests is truly distinguishable. The 0.5 log10 c/mL threshold used to flag a technically meaningful difference was based on the R-Gene kit protocol from Biomerieux (Lyon, France) and previous studies, which outline that a difference of less than 0.5 log10 c/mL within the context of patient follow-up between two quantification results is commonly acknowledged to not be considered significant [23,38].

The thresholds for TTV load classifications and the associated categories utilized for stratified reliability assessment were delineated as follows: negative: no TTV DNA detected; low: small, not quantifiable, amounts of TTV DNA detected <2.4 log10 c/mL; intermediate: moderate amounts of TTV DNA detected, between 2.4 and 3 log10 c/mL; high: large amounts of TTV DNA detected >3 log10 c/mL. To analyze differences between TTV results based on different dilutions, variations in TTV outcomes were evaluated utilizing the Friedman paired non-parametric test. ICC analyses were conducted in the above-mentioned strata, as well as all samples, to gain a generic measurement comparability. Furthermore, the sensitivity and specificity of affirmative test results, with the undiluted test serving as the gold standard, were computed. All TTV values are delineated logarithmically (log10). Additionally, coefficients of variation (CV%) per dilution level (100, 50, 25 µL vs. undiluted) for all measurements and stratified by TTV category, on linear-scale concentrations, were calculated.

To explore factors associated with variability across dilutions, we performed univariable and multivariable linear regression analyses using the specimen-wise standard deviation of TTV measurements as the dependent variable and common demographic and clinical covariates (age, sex, SDAI, disease duration, serostatus, treatment category, glucocorticoid use, and mean log-TTV) as well as storage duration (years) as predictors. The analysis was repeated as a linear mixed-effects model with a patient-level random intercept to account for repeated specimens from the same patient.

No AI tool has been used to prepare this manuscript.

## 3. Results

In 278 serum samples of 128 patients with RA, TTV DNA was measured with decreasing volume (equals stronger dilution) towards testing. In 275 samples, a valid test result throughout all dilution stages could be obtained. The remaining three of the 278 samples (1.1%) yielded a non-evaluable result at one or more dilution stages and were excluded from the corresponding analyses. For some patients, more than one treatment segment was selected for sample selection. The characteristics of the respective patients are outlined in Table 1**.** No correlation between the storage time of samples and TTV load was found (ρ = −0.061; *p* = 0.313).

### 3.1. Effect of Serum Sample Dilution on TTV Quantification

**Figure 1:**
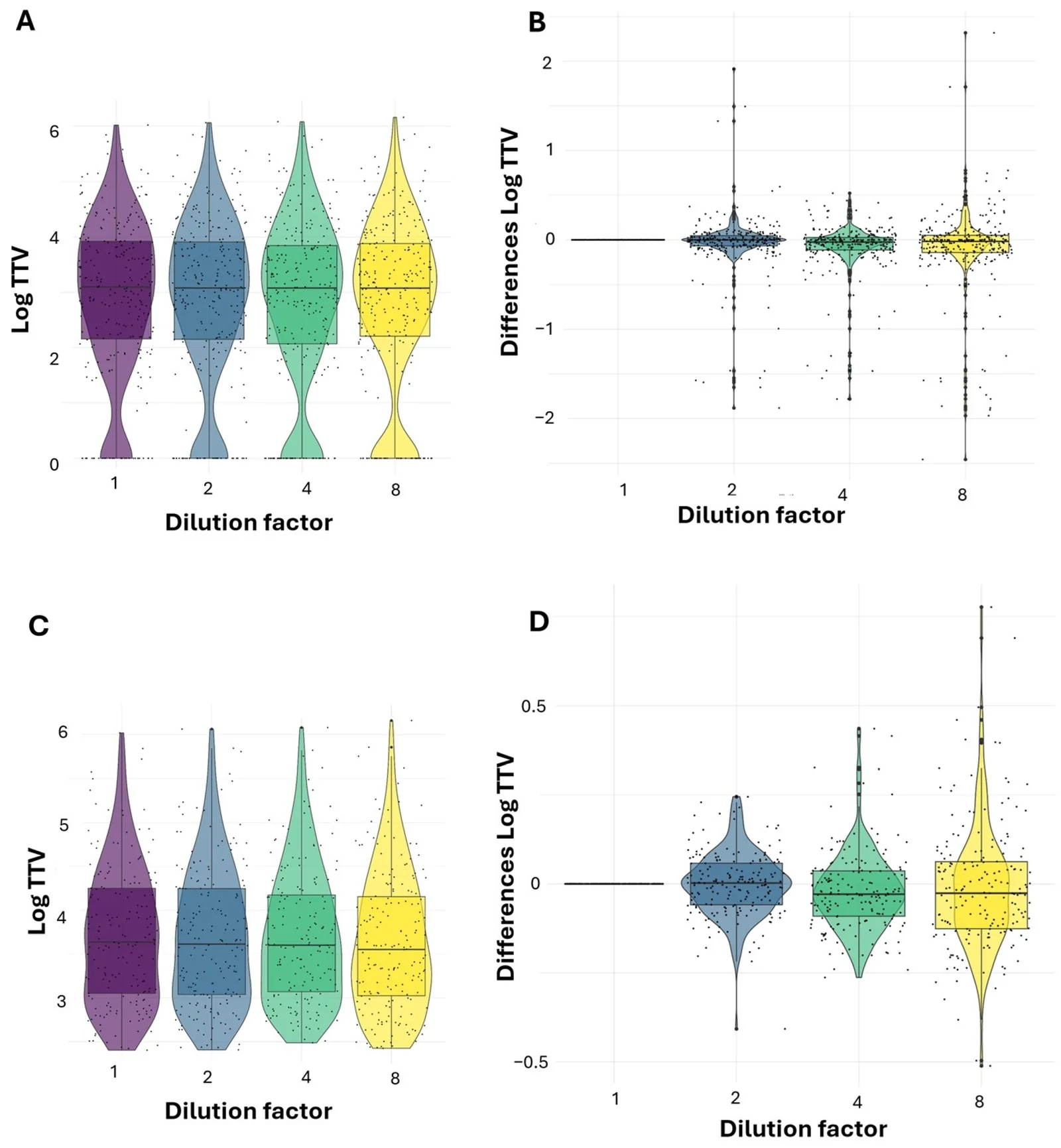
Significant differences in TTV from a 4-fold dilution onwards compared to undiluted tests. (**A**) Violin plot of all log TTV values stratified by dilution factor (1, 2, 4 and 8); (**B**) violin plot of differences between diluted measurements and undiluted (1); (**C**) violin plot of log TTV values by the dilution factor of those above the LLoQ; (**D**) violin plot of differences between diluted and undiluted measurements of those with an undiluted value above the LLoQ.

In the 275 sample dilution series, 32 samples (11.6%) were negative for TTV, 54 (19.6%) had a low viral load, 44 (16%) had an intermediate viral load, and 145 (52.7%) had a high viral load, taking the undiluted testing series as reference. Comparative tests between samples in different dilutions showed statistical differences from a 4-fold dilution onwards, independent of any stratification (Figure 1; 1 vs. 2: *p* = 0.993; 1 vs. 4: *p* < 0.001; 1 vs. 8: *p* < 0.001). TTV values derived from a 2-fold dilution were similar to undiluted samples. As expected, the direction of difference was negative, meaning that higher dilutions show fewer TTV copies. Table 2 outlines measurement values.

As summarized in Table 3, the ICC of all samples and dilutions was 0.95 (95% confidence interval (CI): 0.951 to 0.966). The ICC further on remained similar (0.97; 95% CI: 0.96 to 0.98) when comparing undiluted samples with their 2-fold and 4-fold dilutions, but dropped to 0.94 (95% CI: 0.93 to 0.96) in the comparison to 8-fold dilutions. The same applied to the sensitivity and specificity. The sensitivity declines with higher fold dilution, whereas the CV% (Table 4) increases respectively. The number of differences observed between diluted and undiluted specimens exceeding 0.5 c/mL was equal in the 2- and 4-fold set-up (6.2% and 5.5%) but increased in those 8-fold-diluted to 10.1%. The number of TTV-negative tested samples increased with higher dilution factors (2-fold: 13.5%; 4-fold: 15.6%; and 8-fold: 16.7%).

**Figure 2:**
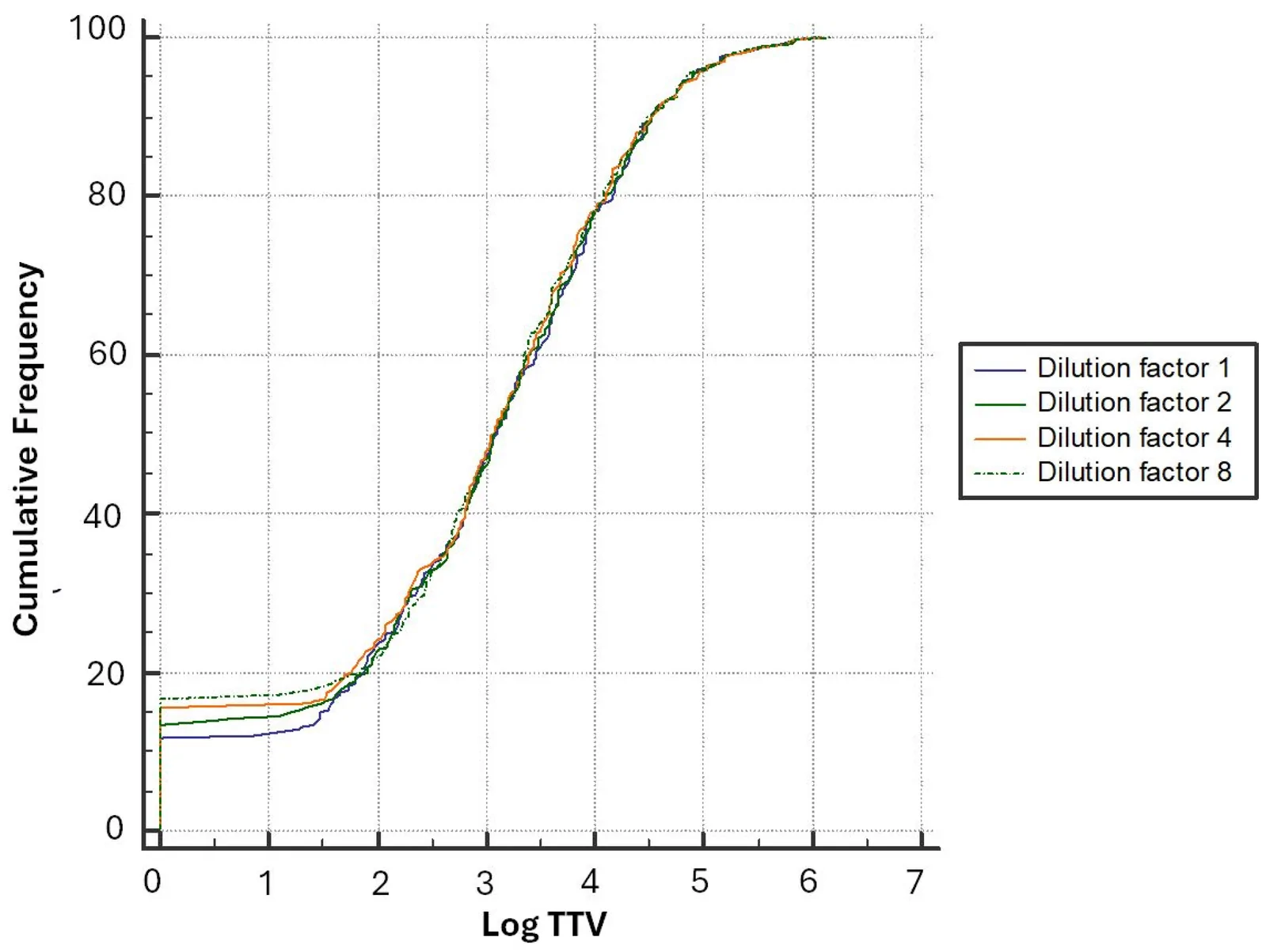
Cumulative frequency plot of log TTV copies/mL separately outlined by dilution factor, highlighting different TTV load measurements in samples below the lower limit of quantification.

A major difference in ICC was seen when stratified by the established cut-offs (Table 3), with lower reliability in samples with lower TTV load. This difference in reliability is supported with the complementing finding of higher CV% in samples with lower TTV load (Table 4). The cumulative frequency plot across all four testing series highlights reduced reproducibility and sensitivity in samples that initially contain low or undetectable loads of TTV (Figure 2).

When stratifying the reliability assessment by patient samples in either REM/LDA or MDA/HDA, the same trend with decreasing ICC was seen when comparing undiluted tests with diluted ones, but the ICC was similar and not dependent on disease activity of RA. In regression analyses, only the mean TTV was significantly associated with variability across dilutions, whereas other clinical or demographic variables were shown to influence the reliability of TTV quantification (Table 5 and Table 6).

### 3.2. Reproducibility of TTV Quantification in Patients with RA

**Figure 3:**
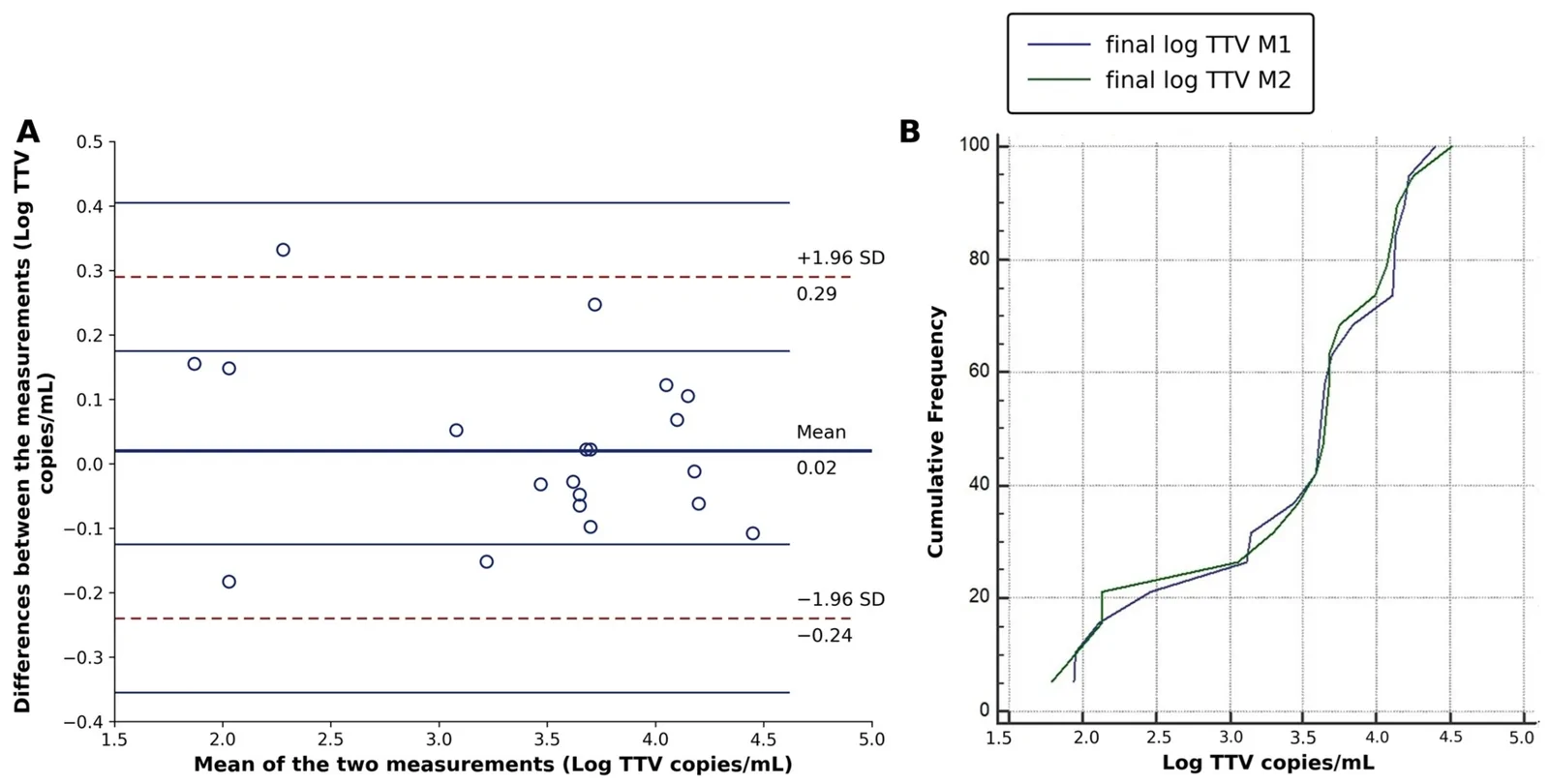
(**A**) Bland–Altman plot outlining the differences between two measurements plotted against the mean of the respective measurements. (**B**) Cumulative frequency of both log TTV measurements, which demonstrates the good agreement between the results of the duplicate measurements. **Abbreviations:** M, measurement; TTV, torque teno virus; log, logarithmic.

Based on 19 samples that were measured twice under the same conditions, the test results stood as proof of strong reproducibility. None of the samples was negative for TTV, three were below the lower level of quantification cut-off and all could be included for reliability testing. The test–retest reliability outlined an ICC of 0.986 (95% CI: 0.97 to 0.99). The Bland–Altman plot depicted only minor absolute differences in duplicates, which were all below a technical level of importance of 0.5 c/mL (Figure 3A). This coincides with an SDD of 0.25 c/mL. This agreement between the values is supported by the cumulative frequency plot of the two sets of TTV measurements (Figure 3B).

## 4. Discussion

This is the first study investigating the accuracy of TTV quantification in patients with RA. Most importantly, the study proves the high consistency and reproducibility of TTV quantification based on the excellent ICC and only a small SDD of 0.25 c/mL. The SDD was therefore smaller than technically relevant changes of log 0.5 c/mL and above.

The ability to accurately quantify TTV load is foundational for its clinical utility, particularly when considering its use in monitoring immunomodulatory effects of DMARDs, where current biomarkers of immunocompetency are lacking. Since the effect of different DMARDs on TTV replication is not well understood, we subjected a representative set of serum samples from patients with RA on different DMARD therapies and investigated whether a more parsimonious approach of sample utilization might be feasible to investigate TTV as a biomarker in larger cohorts in the future. Our study highlights the significant impact of sample dilution on TTV quantification. The overall ICC for all samples and dilutions was 0.95, which is considered excellent; however, a notable decrease in ICC was observed with higher fold dilutions, specifically dropping from 0.97 to 0.94 for an 8-fold dilution compared to undiluted samples. The number of TTV-negative tested samples also increased with higher dilution factors, suggesting that dilution can lead to false negatives or underestimation of viral load. This effect is particularly pronounced in samples with initially low or undetectable TTV load, where reproducibility and sensitivity will be compromised. Because the ICC depends on the between-specimen variance, the high pooled ICC partly reflects the wide dynamic range of TTV load in this cohort; we therefore complemented it with category-stratified ICCs, which reveal markedly reduced reliability at low and undetectable loads (ICC of 0.16 for negative and 0.46 for sub-LLoQ specimens). The association of measurement variability with lower TTV load, but not with demographic or clinical variables, is consistent with a technical rather than biological origin: dispersion increases near the lower limit of quantification. Accordingly, TTV quantification is unreliable at low loads, and any prospective application should be cautious with low quantifiable loads.

When comparing TTV load in our population of RA patients (median: 3.1 log10 c/mL; IQR: 1.764) to those patients treated with calcineurin-inhibitors (median: 7.1 log10 c/mL; IQR: 5.3–8.9) due to transplantation [39], we generally observe lower TTV values, indicating potentially a less severe or more targeted immunosuppression. In contrast, TTV load in blood donors revealed a median TTV load of 2.3 log c/mL [23]. Thus, a cut-off of 3.0 log10 c/mL for labelling samples with a high TTV category in patients with RA can pragmatically be considered. TTV might only be of value for monitoring RA patients, if the applied immunomodulatory agents indeed influence TTV replication, implying that this sort of functional immune-monitoring might not be feasible with low TTV loads. This can be regarded as a limiting factor since several of our samples showed results below the lower limits of quantification of 2.4 log10 c/mL, which are at risk of unstable reproducibility, outlined by an ICC of 0.46. The poorer sensitivity of TTV detection might however not be a problem in future clinical application, since moderate to high loads might only be relevant in potential future risk calculation for balancing beneficial effects and risks of immunosuppressive treatment.

Modifying factors of TTV load in patients with RA are not yet fully explored; however, a previous study investigating the utility of TTV in a trial randomizing MTX-insufficient responders to either rituximab, infliximab, abatacept or tocilizumab provided insights for testing further hypotheses [21]. An increase in TTV during the first 3 months of treatment could be seen in all patients independent of the compound besides those receiving tocilizumab. A decrease in disease activity was similar in all four treatment groups, which indicates that the type of immunomodulation is the prominent influential factor on viral load. TTV load in our data did not differ between the two disease activity groups.

Further aspects that need to be taken into account in the interpretation of TTV load, but not in terms of reproducibility, might be ethnicity and geographical differences [5], despite our centre representing a patient collective of a diverse European municipality. The group of patients without DMARD therapy but exhibiting high and low disease activity without treatment adjustment could have been larger to represent TTV measurements without treatment as an influential factor. Since RA is a chronic condition necessitating long-term DMARD treatment, periods without treatment harbour the risk of flares and structural damage accrual [40,41,42], thus limiting studies in this population.

Other factors to consider in interpreting our results are the single-centre analysis using a single commercial qPCR assay, evaluating analytical reproducibility and minimal serum input rather than the clinical validity or utility of TTV. The assay used in our study has, however, been validated in large inter-laboratory comparisons including 13 centres [43] and showed good concordance compared to an in-house PCR [44]. Our samples had been stored for up to 18 years under stable conditions at −80 degrees Celsius, and although anellovirus DNA is considered stable under standardized storage, a residual effect of storage duration cannot be fully excluded. Specimen selection was purposive rather than random, and several patients contributed more than one specimen. The dedicated test–retest set was modest (*n* = 19) but sufficient and weighted towards higher loads. Hence, this cohort that underwent TTV measurement of duplicated samples to determine test–retest reliability might even provide stronger estimates if saturated with more granularity in TTV viral load heterogeneity, since the majority of samples revealed a TTV above 3 log10 c/mL. Reproducibility and sensitivity were reduced at low and undetectable loads, which constrains any future application of TTV monitoring to patients above the lower limit of quantification (>2.4 log10 c/mL).

In the field of rheumatology, TTV is a novel biomarker. Due to the heterogeneity of the disease and its treatment options, large collaborative investigations are warranted. For the execution of multi-centre studies, optimal planning and resource allocation is of high importance to reach research objectives with an ideal trade off in means. Biospecimen volume is of great interest to all investigators, underlining the need for our study. Therefore, a balance must be struck between sample conservation and analytical reliability. For clinical applications, especially in RA where TTV is being explored as a prognostic biomarker, consistent and accurate quantification is paramount. Our results indicate that reducing the standard volume of 200 µL of serum by half (100 µL) does not compromise test accuracy. A further dilution by a factor of 4 (50 µL) might be feasible in case of particularly valuable samples, whose inclusion into the investigation is crucial and would otherwise not be possible. This recommendation is intended for large-scale cohort screening in the quantifiable range; a small proportion of near-threshold specimens may be reclassified after dilution, and sensitivity declines close to the lower limit of quantification, so reduced input should be applied cautiously to low-load samples.

## 5. Conclusions

This is the first study to assess the stability and reproducibility of TTV testing in patients with RA. First, a reduction in sample volume by 50% to 100 µL of human serum can be recommended, as we did not find a negative impact on TTV measurement results. In addition, assessments of duplicates showed a high reproducibility of results. Finally, the reliability of measurement of TTV was not influenced by disease activity, so dilution can therefore be applied to all groups of RA patients, taking into account that sensitivity might be lower in patients with a TTV load below the lower limit of quantification. In summary, TTV measurement will potentially allow, with small amounts of biological material and small efforts, to generate highly complementary information to currently used biomarkers, although its clinical validity and utility for guiding treatment decisions remain to be established in dedicated clinical studies.

## Figures and Tables

**Table 1:** Descriptive overview of the cohort. Values are in mean ±SD, unless indicated otherwise.

Characteristic	CDAI < 8 N = 139 ^1^	CDAI > 12 N = 139 ^1^	Test–Retest N = 19 ^1^
**Demographic**			
Female %	110 (79%)	110 (79%)	10 (59%)
Age	59 (12)	60 (12)	50 (19)
Time since DMARD start (months)	25 (21)	34 (35)	2 (3)
Time between samples (months)	23 (27)	23 (27)	
Number of treatment segments	5.5 (4.0)	5.5 (4.0)	1.2 (0.4)
Glucocorticoids (yes)	56 (42%)	67 (52%)	9 (64%)
**Clinical**			
CCP-positive	96 (71%)	96 (72%)	10 (63%)
RF-positive	82 (61%)	82 (62%)	7 (47%)
CRP (mg/dL)	0.52 (0.99)	0.80 (1.27)	2.97 (7.41)
SJC28	0.9 (1.1)	3.9 (2.8)	4.4 (3.6)
TJC28	0.8 (1.0)	5.7 (4.4)	5.1 (4.9)
DAS28	2.25 (0.60)	3.95 (0.73)	3.75 (1.09)
CDAI	4.5 (2.4)	17.0 (5.7)	15.1 (6.6)
SDAI	5.0 (2.7)	17.8 (6.2)	16.4 (8.0)
**DMARDs**			
Methotrexate	20 (14%)	20 (14%)	11 (58%)
Leflunomide	20 (14%)	20 (14%)	0
TNFi	40 (29%)	40 (29%)	0
Abatacept	10 (7.2%)	10 (7.2%)	0
Tocilizumab	18 (13%)	18 (13%)	0
Rituximab	20 (14%)	20 (14%)	0
Other csDMARD	0 (0%)	0 (0%)	2 (11%)
No DMARD treatment	11 (7.9%)	11 (7.9%)	6 (32%)
**TTV**			
log TTV undiluted	2.94 (1.49)	2.90 (1.39)	
log TTV dilution factor 2	2.90 (1.54)	2.87 (1.43)	3.44 (0.79)
log TTV dilution factor 4	2.84 (1.57)	2.82 (1.46)	
log TTV dilution factor 8	2.81 (1.59)	2.82 (1.48)	

**Abbreviations:** RF, rheumatoid factor; CCP, cyclic citrullinated peptide; CDAI, clinical disease activity index; DMARD, disease-modifying anti-rheumatic drug; cs, conventional synthetic; TNF, tumour necrosis factor; SJC, swollen joint count; TJC, tender joint count; TTV, torque teno virus. ^1^ *n* (%); Mean (SD).

**Table 2:** Descriptive statistics of log TTV values stratified by dilution factor and TTV load in undiluted sample.

**All Samples**			
**Dilution**	**Count**	**Mean**	**SD**
Undiluted	275	2.909	1.438
2	275	2.875	1.482
4	275	2.822	1.517
8	275	2.816	1.534
**TTV > 2.4 c/mL in undiluted test**			
Undiluted	189	3.696	0.782
2	189	3.687	0.788
4	189	3.660	0.798
8	189	3.640	0.839
**TTV > 3 c/mL in undiluted test**			
Undiluted	145	3.983	0.659
2	145	3.968	0.675
4	145	3.940	0.690
8	145	3.936	0.686

**Abbreviations:** SD, standard deviation; TTV, torque teno virus; c/mL, copies per mL.

**Table 3:** Intra-class correlation (ICC) testing different pre-conditions. Sensitivity and specificity of 2-fold-, 4-fold- and 8-fold-diluted samples for TTV measurement.

Groups	Count	ICC	95% CI	Sensitivity	Specificity
Undiluted vs. 2	275	0.9727	0.966–0.978	96.75	90.62
Undiluted vs. 4	275	0.9759	0.967–0.982	95.53	100.00
Undiluted vs. 8	275	0.9478	0.933–0.959	93.50	93.75
TTV-negative	32	0.1688	0.018–0.371		
TTV < LLoQ	54	0.464	0.327–0.604		
TTV = 2.4–3.0 c/mL	44	0.4398	0.288–0.599		
TTV > 3.0 c/mL	145	0.9868	0.983–0.990		

**Abbreviations:** ICC, intra-class-correlation; CI, confidence interval; TTV, torque teno virus; LLoQ, lower limit of quantification, c/mL, copies/millilitre.

**Table 4:** Coefficient of variation testing different pre-conditions in 2-fold-, 4-fold- and 8-fold-diluted samples for TTV measurement.

Dilution	TTV Groups	Mean CV%	Median CV%	SD CV%	N
Undiluted vs. 2	All	18.34	9.96	28.66	275
Undiluted vs. 4	All	20.86	11.54	28.20	275
Undiluted vs. 8	All	28.87	17.11	34.65	275
Undiluted vs. 2	TTV-negative	12.48	0.00	39.44	32
Undiluted vs. 4	TTV-negative	0.00	0.00	0.00	32
Undiluted vs. 8	TTV-negative	8.63	0.00	33.95	32
Undiluted vs. 2	TTV < LLoQ	46.74	29.71	43.76	54
Undiluted vs. 4	TTV < LLoQ	54.73	40.89	43.24	54
Undiluted vs. 8	TTV < LLoQ	71.07	61.01	45.31	54
Undiluted vs. 2	TTV = 2.4–3.0 c/mL	16.13	13.13	14.73	44
Undiluted vs. 4	TTV = 2.4–3.0 c/mL	21.34	16.16	15.97	44
Undiluted vs. 8	TTV = 2.4–3.0 c/mL	34.15	32.93	25.71	44
Undiluted vs. 2	TTV > 3.0 c/mL	9.84	7.81	9.14	145
Undiluted vs. 4	TTV > 3.0 c/mL	12.80	9.31	12.97	145
Undiluted vs. 8	TTV > 3.0 c/mL	15.99	13.99	12.35	145

**Abbreviations:** CV%, coefficient of variation; SD, standard deviation; TTV, torque teno virus; LLoQ, lower limit of quantification, c/mL, copies/millilitre.

**Table 5:** Regression model exploring sample-wise standard deviations of TTV measurements across dilutions.

	Univariable		Multivariable	
Factor	Estimate	*p*-Value	Estimate	*p*-Value
Age	−0.001	0.364	0.000	0.879
Sex	−0.004	0.907	0.043	0.256
SDAI	0.000	0.922	0.001	0.694
Disease duration (months)	0.000	0.054	0.000	0.135
Seropositivity	0.044	0.156	0.021	0.528
DMARD treatment category	0.000	0.975	−0.004	0.608
Glucocorticoids (yes/no)	−0.026	0.400	−0.033	0.283
Storage time (years)	0.000	0.214	0.000	0.258
Mean log-TTV	−0.071	<0.001	−0.072	<0.001

**Abbreviations:** SDAI, Simplified Disease Activity Index; TTV, torque teno virus.

**Table 6:** Regression model exploring sample-wise standard deviations of TTV measurements across dilutions, in a mixed-effects model with a patient-level random intercept.

	Univariable		Multivariable	
Factor	Estimate	*p*-Value	Estimate	*p*-Value
Age	−0.001	0.433	0.000	0.854
Sex	−0.016	0.739	0.035	0.427
SDAI	0.000	0.753	0.001	0.622
Disease duration (months)	0.000	0.155	0.000	0.155
Seropositivity	0.030	0.403	0.016	0.642
DMARD treatment category	0.000	0.972	−0.004	0.666
Glucocorticoids (yes/no)	−0.019	0.531	−0.027	0.356
Storage time (years)	0.000	0.336	0.000	0.359
Mean log-TTV	−0.070	<0.001	−0.071	<0.001

**Abbreviations:** SDAI, Simplified Disease Activity Index; TTV, torque teno virus.

## Data Availability

The original contributions presented in this study are included in the article. Further inquiries can be directed to the corresponding author.

## Abbreviations

The following abbreviations are used in this manuscript:

TTV	Torque Teno Virus
RA	Rheumatoid Arthritis
SDD	Smallest Detectable Difference
ICC	Intra-Class Correlation
SD	Standard Deviation
CV%	Coefficient of Variation
LLoQ	Lower Limit of Quantification
DMARD	Disease-Modifying Anti-Rheumatic Drugs
EULAR	European Alliance of Associations for Rheumatology
TNFi	Tumour Necrosis Factor inhibitor
µl	Microliter
CDAI	Clinical Disease Activity Index
SDAI	Simplified Disease Activity Index
PCR	Polymerase Chain Reaction
IQR	Interquartile Range
RF	Rheumatoid Factor
CCP	Cyclic Citrullinated Peptide
SJC	Swollen Joint Count
TJC	Tender Joint Count
CRP	C-Reactive Protein
c/mL	Copies per Millilitre
CI	Confidence Interval
REM	Remission
LDA	Low Disease Activity
MDA	Moderate Disease Activity
HDA	High Disease Activity
